# An Alternative Suite of Universal Primers for Genotyping in Multiplex PCR

**DOI:** 10.1371/journal.pone.0092826

**Published:** 2014-03-21

**Authors:** Cheng Ge, Yu-Nan Cui, Peng-Yu Jing, Xiao-Yue Hong

**Affiliations:** Department of Entomology, Nanjing Agricultural University, Nanjing, Jiangsu, China; Duke University, United States of America

## Abstract

The universal primer three-primer approach can dramatically reduce the cost when genotyping the microsatellites. One former research reported four universal primers that can be used in singleplex and multiplex genotyping. In this study, we proposed an alternative suite of universal primers with four dyes for genotyping 8–12 loci in one single run. This multiplex method was tested on *Tetranychus truncatus*. Published microsatellite loci of *T. kanzawai*, *Frankliniella occidentalis* and *Nilaparvata lugens* were modified as needed and also tested. The robustness of the method was confirmed by comparing with singleplex using multiple fluorophores and genotyping two populations of *T. truncatus*. This method showed lower signal strength than the singleplex three-primer system, but it was still sufficient to determine the fragment length. The cost of such a project can be reduced dramatically when many loci of different species are involved. In this way, laboratories performing population genetic analyses or studying several different species may benefit from the use of this cost-effective protocol.

## Introduction

Microsatellites were first genotyped by Litt and Luty [1]. There are now a number of methods available for the identification of SSR alleles. Methods based on radioactive isotopes and silver staining have been almost completely replaced by the more rapid and precise capillary electrophoresis fluorescence detection system, which has revolutionized the field. Some 90 out of 100 articles relying on SSRs published in 2009 and 2010 in the journal *Molecular Ecology* involved subjects genotyped using this automatic capillary electrophoresis system [2].

In capillary electrophoresis systems, forward or reverse primer of one specific locus was usually directly labeled with a fluorescent tag. Then this fluorescence is incorporated into PCR products, which can be detected in the capillary sequencer. Then the length of the PCR products can be analyzed. However, the use of this method is limited because the fluorescent dyes are very expensive and the cost increases considerably when a large number of loci are involved. Meanwhile, an economical approach was proposed and improved [3]–[7], which have been cited for more than 1300 times. The universal primer three-primer approach is based on a universal fluorescent primer (M13) which is identical to the sequence added at 5′ end of each forward primer. During the latter PCR cycles, this universal fluorescent primer hybridizes with the complementary region generated in the early PCR cycles. In this way, instead of synthesizing fluorescent-labeled primer for each SSR locus, only one universal labeled primer must be fluorescent.

Theoretically, this dye-labeled universal primer method can be multiplexed [6]. However, the universal primer three-primer approach limits multiplexing if loci sizes do not overlap. Although the M13 sequence is the most widely used tail, any sequence that is not homologous to the target genome could be used. Missiaggia and Grattapaglia tested *Eucalyputs* DNA samples using three universal tags DYS437, D8S1132 and D12S1090, which are derived from human microsatellites [8]. Moreover, Blacket *et al*. demonstrated that four universal primers can be combined with four fluorophores to co-amplify multiple loci via multiplex PCR [9]. This strategy, called strategy III-Multiplex with multiple fluorophores, is highly cost-effective method and has been proven to be feasible [10]–[13].

Here the concept and methodology of strategy III described by Blacket *et al* were kept [9]. The simultaneous use of another four universal primers combined with four fluorophores to genotype 8–12 loci in a single PCR run is proposed here. The effectiveness of the new set of universal primers in multiplex genotyping was tested for the detection of microsatellites of spider mites *Tetranychus truncatus* and further validated by genotyping two populations of *T. truncatus*. Moreover, published microsatellite loci of *T. kanzawai*, *Frankliniella occidentalis* and *Nilaparvata lugens* were modified as needed for universality test.

## Materials and Methods

### Mite collection and identification

Mites were collected in 2011 and 2012 from five provinces in China (Table 1). The mites were reared on detached bean leaves (*Phaseolus vulgaris*) at 25°C in a 16 h light/8 h dark cycle.

**Table 1:** Collection information for samples used in this study.

Population name	Site	Location	Species	Sampling date	No. individuals used	Host
BC	Baotou	40°39′N,109°50′E	*T. truncatus*	Sep. 2011	4	*Phaseolus vulgaris*
SQ	Shenyang	41°49′N, 123°33′E	*T. truncatus*	Sep. 2011	50	*Solanum melongena*
XZ	Xuzhou	34°44′N,116°56′E	*T. truncatus*	Jul. 2012	42	*Gossypium hirsutum*
XN	Xining	36°29′N,101°34′E	*T. kanzawai*	Sep. 2011	4	*Livistona chinensis*
PZ	Pengze	29°51′N,116°35′E	*T. kanzawai*	Jul. 2012	4	*G. hirsutum*
HB	Harbin	45°45′N,126°38′E	*F. occidentalis*	Jul. 2010	4	*Tagetes erecta*
DL	Dali	25°36′N,100°15′E	*F. occidentalis*	Jul. 2011	4	*Canna indica*
SY	Sanya	29°51′N,116°35′E	*N. lugens*	Aug. 2013	4	*Oryza sativa*
GL	Guilin	29°51′N,116°35′E	*N. lugens*	Aug. 2013	4	*O. sativa*

The identification of spider mites has long been an issue because different species are very similar. In one previous study, both a morphological approach based on the shape of the aedeagus and a molecular approach based on the internal transcribed spacer (ITS) region sequence were used to confirm the identities of spider mite individuals, which proved to be accurate and efficient [14]. This method was also used in the present study.

No specific permits were required for the study. (a) No specific permissions were required for the mite collections because the mites are pests on crops; (b) The location is not privately-owned; (c) The study did not involve endangered or protected species.

### DNA extraction and primer preparation

Female *T. kanzawai* were directly picked from detached bean leaves and then washed with Milli-Q water. Genomic DNA was extracted using a DNeasy tissue kit (Qiagen, U.S.) following the protocol described by Tsagkarakou *et al*. [15]. The DNA of *T. truncates* was extracted in 2012 using the same protocol and stored in −20°C. The DNA samples of *F. occidentalis* and *N. lugens* were provided by our colleagues (collection information see Table 1). The quality of all DNA samples was estimated using a NanoDrop ND-1000 spectrophotometer.

Twelve primer pairs (Table 2) were selected for amplification of the SSR loci of *T. truncates* with the help of Multiplex Manager 1.2 [14], [16]. Each specific forward primer was connected with one of the 5′ universal primer sequence tails which were T7 (5′-TAATACGACTCACTATAGGG), M13 (5′-TGTAAAACGACGGCCAGT), M13R (5′-CAGGAAACAGCTATGACC) or AP2 (5′-CTATAGGGCACGCGTGGT) (Table 2). The stand alone universal primer M13, AP2, M13R and T7 were fluorescent-labeled with FAM, VIC, NED and PET respectively. All primers were ordered from Life Technologies and dissolved in TE (10 mM Tris, 1 mM EDTA, pH 8.0) to produce a 20 μM stock solution for fluorescent-labeled universal primer and locus-specific reverse primer and a 5 μM stock solution of locus-specific forward primer with universal tail (moles of labeled forward primer: reverse primer: dye-labeled universal primer = 1∶4∶4 [6]). Primer mix is here designated P_S_ (three primers were mixed for each specific locus and are collectively called P_S_, as in P_TUFG87_). P_A_, P_B_, P_C_, P_A+B_ and P_A+B+C_ were also prepared. They contained all corresponding P_s_s (see Table 2) at equal volume and then stored in small aliquots at −20°C in the dark.

**Table 2:** Universal primer, dye and group of microsatellite primers of *Tetranychus* species used in this study.

Species	Locus(P_S_)	Universal primer added to forward primer	Dye	Group
*T. truncatus*	TUFG87	M13	FAM	P_A_
	TUFG57	AP2	VIC	
	TUFG127	M13R	NED	
	TUFG130N	T7	PET	
*T. truncatus*	TUFG177	M13	FAM	P_B_
	TUFG55	AP2	VIC	
	TUFG146	M13R	NED	
	TUFG147N	T7	PET	
*T. truncatus*	TUFG33	M13	FAM	P_C_
	TUFG43	AP2	VIC	
	TUFG67	M13R	NED	
	TUFG158	T7	PET	
*T. kanzawai*	TkMS002	M13	FAM	P_E_
	TkMS010	AP2	VIC	
	TkMS011	M13R	NED	
	TkMS014	T7	PET	
*T. kanzawai*	TkMS006	M13	FAM	P_F_
	TkMS013	AP2	VIC	
	TkMS015	M13R	NED	

P_A+B_, all the P_s_s in primer mix P_A_ and P_B_ were pooled; P_A+B+C_, all the P_s_s in primer mix P_A_, P_B_ and P_C_ were pooled.

### Genotyping

The usefulness of these four universal primers was tested with eight *T. truncatus* samples from population XZ and BC in three levels: singleplex PCR, multiplex PCR in which each dye labeled one locus and the multiplex PCR in which each dye labeled more than one locus. At level I, PCR was carried out in 5 μL volumes using an Applied Biosystems Veriti Thermal Cycler (Applied Biosystems). Each assay contained 2.1 μL RNase-free water, 1 μL template DNA, 0.15 μL P_S_ and 1.75 μL Type-it Microsatellite PCR Master Mix (2X, Qiagen). At level II and III, PCR was performed in a final volume of 5 μL containing 1.95 μL RNase-free water, 1 μL template DNA, 0.3 μL P_A_/P_B_ (level II) or P_A+B_/P_A+B+C_ (level III) and 1.75 μL Type-it Microsatellite PCR Master Mix (2X, Qiagen). At all three levels, PCR was performed on the same DNA samples in the same 96 well plate simultaneously under the same cycling conditions: an initial activation step for 5 min at 95°C; 8 cycles of 95°C for 30 s, 57°C for 90 s, 72°C for 30 s, followed by 14 cycles of 95°C for 30 s, annealing at 57–50°C decreased by 0.5°C per cycle for 90 s, 72°C for 30 s, and then 12 cycles of 95°C for 30 s, 52°C for 90 s, 72°C for 30 s and a final extension at 60°C for 30 min.

At level I, PCR products of the same DNA samples were all mixed and pooled within a well. The 1.0 μL of the mixture of level I or 1.0 μL PCR product in level II or III was added to 10 μL formamide and then electrophoresed on the ABI 3130 capillary sequencer (Applied Biosystems) along with the GeneScan-500 LIZ size standard. The data were collected automatically and alleles were called using GeneMapper v4.0 (Applied Biosystems).

### Method further validation

The usefulness of multiplex PCR with these four alternative universal primers was validated through the genotyping of two populations of *T. truncatus* and microsatellites of other species which have been published. Two populations of *T. truncatus* were amplified by primer mix P_A+B_. The PCR procedure and condition were the same as above. For further validation, seven microsatellites primers that used in *T. kanzawai* were chosen [17]. In the original paper, one primer in each locus was modified with fluorescent dye 6-FAM, HEX, or TAMRA. Therefore, these primers were directly modified as shown in Table 2 to accommodate our protocol. PCR mixtures were prepared as above with the following profile: 95°C for 5 min; 35 cycles of 95°C for 30 s, 52°C for 90 s, and 72°C for 30 s; and a final elongation at 60°C for 30 min. The genotyping procedure was the same as of the one used for *T. truncates*.

Further, ten and twelve microsatellite loci with null allele frequency<0.1 were selected for genotyping eight samples of *F. occidentalis* [18] and *N. lugens* [19] respectively. The primers of each locus were modified as shown in Table 3. PCR mixtures were prepared and genotyped as above. PCR profile for *F. occidentalis* was 5 min at 95°C, 32 cycles of 95°C for 30 s, 52°C for 90 s, 72°C for 30 s, followed by a final extension at 60°C for 30 min. PCR profile for *N. lugens* was 5 min at 95°C, 12 cycles of 95°C for 30 s, annealing at 58–52°C decreased by 0.5°C per cycle for 90 s, 72°C for 30 s, followed by 20 cycles of 95°C for 30 s,52°C for 90 s, 72°C for 30 s, and then 60°C for 30 min.

**Table 3:** Universal primer and dye of microsatellite primers of *F. occidentalis* and *N. lugens* used in this study.

Species	Locus	Universal primer*	Dye	Species	Locus	Universal primer*	Dye
*F. occidentalis*	WFT20	M13	FAM	*N. lugens*	NL22	M13R	NED
	WFT28	T7	PET		NL41	M13	FAM
	WFT37	M13	FAM		NL74	M13R	NED
	WFT51	M13	FAM		NL121	T7	PET
	WFT64	AP2	VIC		NL122	AP2	VIC
	WFT66	M13R	NED		NL140	M13	FAM
	WFT83	T7	PET		NL157	AP2	VIC
	WFT98	T7	PET		NL158	AP2	VIC
	WFT104	M13R	NED		NL161	T7	PET
	WFT108	AP2	VIC		NL162	T7	PET
					NL167	M13	FAM
					NL177	M13R	NED

*Universal primer added to corresponding forward primer.

### Data analysis

Only genotyping data from populations of *T. truncates* were analyzed as follows. Null allele frequencies were determined with Micro-Checker version 2.2.3 with the Brookfield's null allele estimator 2 [20], [21]. Genotype errors such as stuttering or large allelic dropout were also detected using Micro-Checker. Genepop 4.0.10 was used to search for deviations from Hardy–Weinberg equilibrium (HWE) at each locus/population combination using Fisher's exact tests [22]. The population genetic diversity parameters of the population such as total alleles per locus, observed heterozygosity and expected heterozygosity were calculated in GenAlEx 6.5 [23]. Polymorphism information content (PIC) was calculated using Cervus version 3.0 and inbreeding coefficient (*F*_IS_) with the FSTAT 2.9.3.2 [24], [25].

## Results and Discussion

Here the methodology described by Schuelke and Blacket *et al*. were inherited and extended by proposing another suite of universal primers for simultaneously genotyping 8–12 loci in a single run. [6], [9]. In addition, direct comparisons between singleplex and multiplex PCR were presented and different levels of multiplex genotyping were evaluated. This significantly reduces the time and cost required for medium-throughput microsatellite genotyping experiments. Twelve SSR markers from a previous paper served as volunteers to genotype *T. truncatus* DNA samples. The universality of the method was tested by modifying existing primers of microsatellite loci then genotyping *T. kanzawai*, *F. occidentalis* and *N. lugens*. The method was tested further by genotyping two populations of *T. truncatus*.

**Figure 1:**
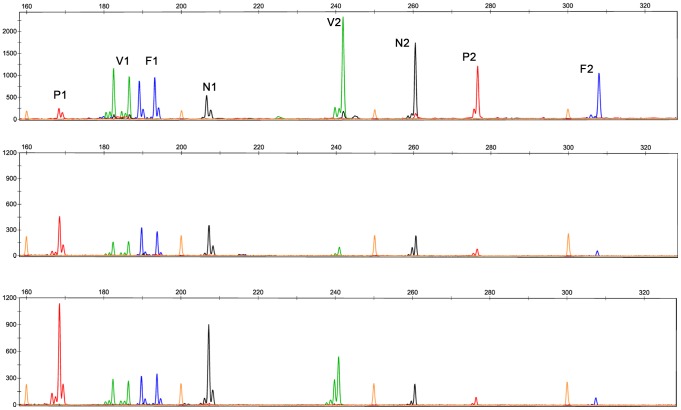
Multiplex PCR results of *T. truncatus* BC02. Three panels refer to singleplex, samples amplified by P_A_ and P_B_ separately then were pooled for capillary loading and samples amplified by P_A+B_ respectively. P1: TUFG130N. V1: TUFG55. F1: TUFG177. N1: TUFG127. V2: TUFG57. N2: TUFG146. P2: TUFG147N. F2: TUFG87.

**Figure 2:**
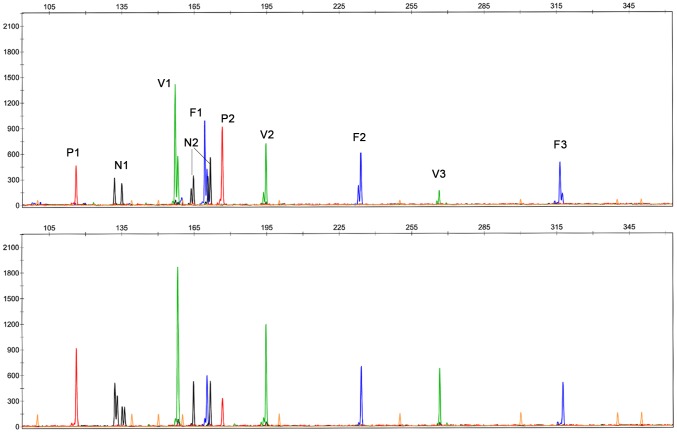
Multiplex PCR results of *F. occidentalis* HB04. Two panels refer to singleplex and sample amplified by ten loci in one multiplex PCR respectively. P1: WFT28. N1: WFT66. V1: WFT108. N2: WFT104. F1: WFT20. P2: WFT98. V2: WFT83. F2: WFT83. V3: WFT64. F3: WFT51.

**Figure 3:**
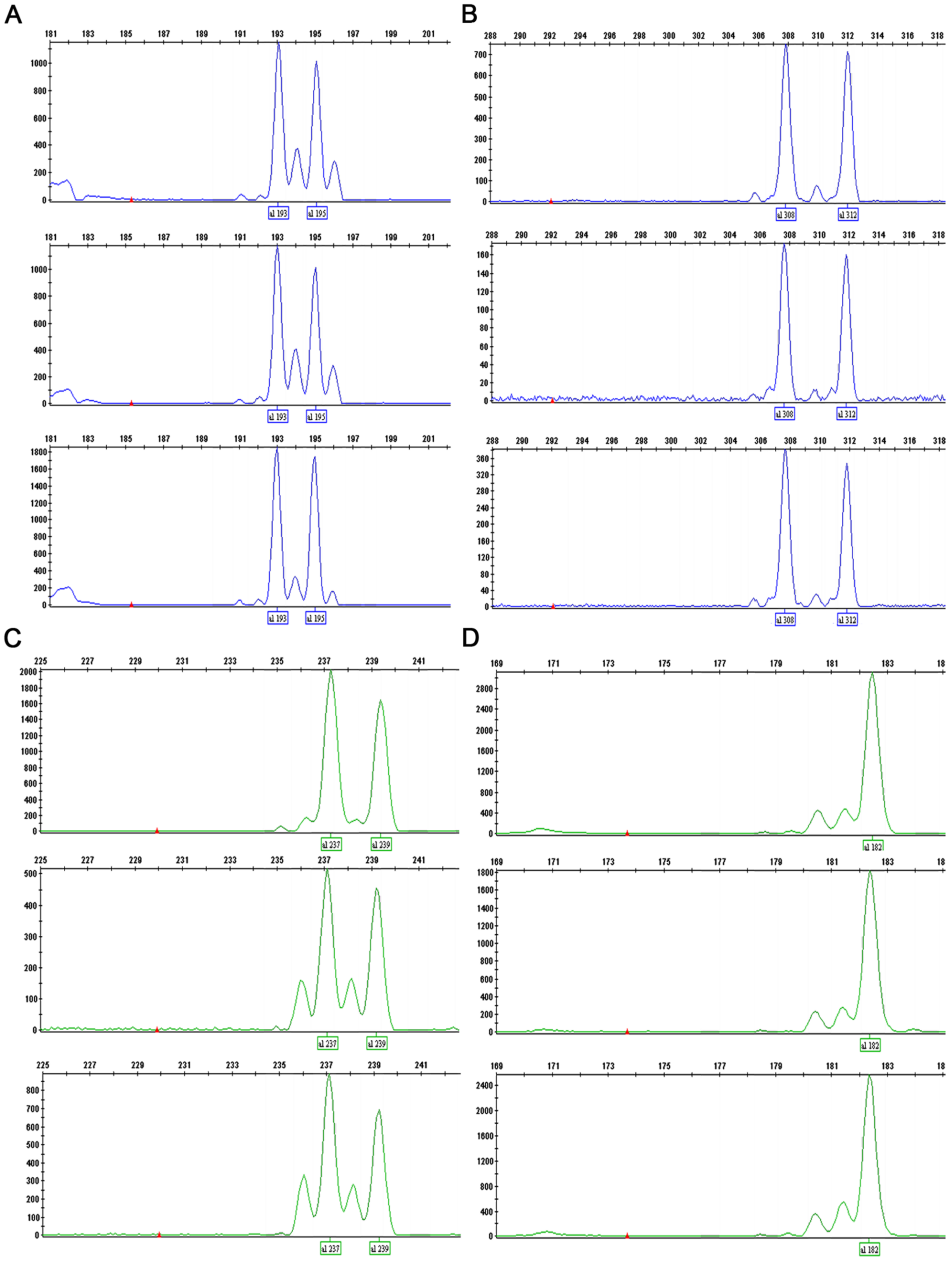
Microsatellite profiles of DNA XZ04 amplified by four loci using three methods generated on a 3130 capillary sequencer. **a**) locus TUFG177; **b**) locus TUFG87; **c**) locus TUFG57; **d**) locus TUFG55. Each part of figure contains three screenshots, which here represent singleplex, samples amplified by P_A_ and P_B_ then were pooled for capillary loading and samples amplified by P_A+B_ using the method described herein.

**Figure 4:**
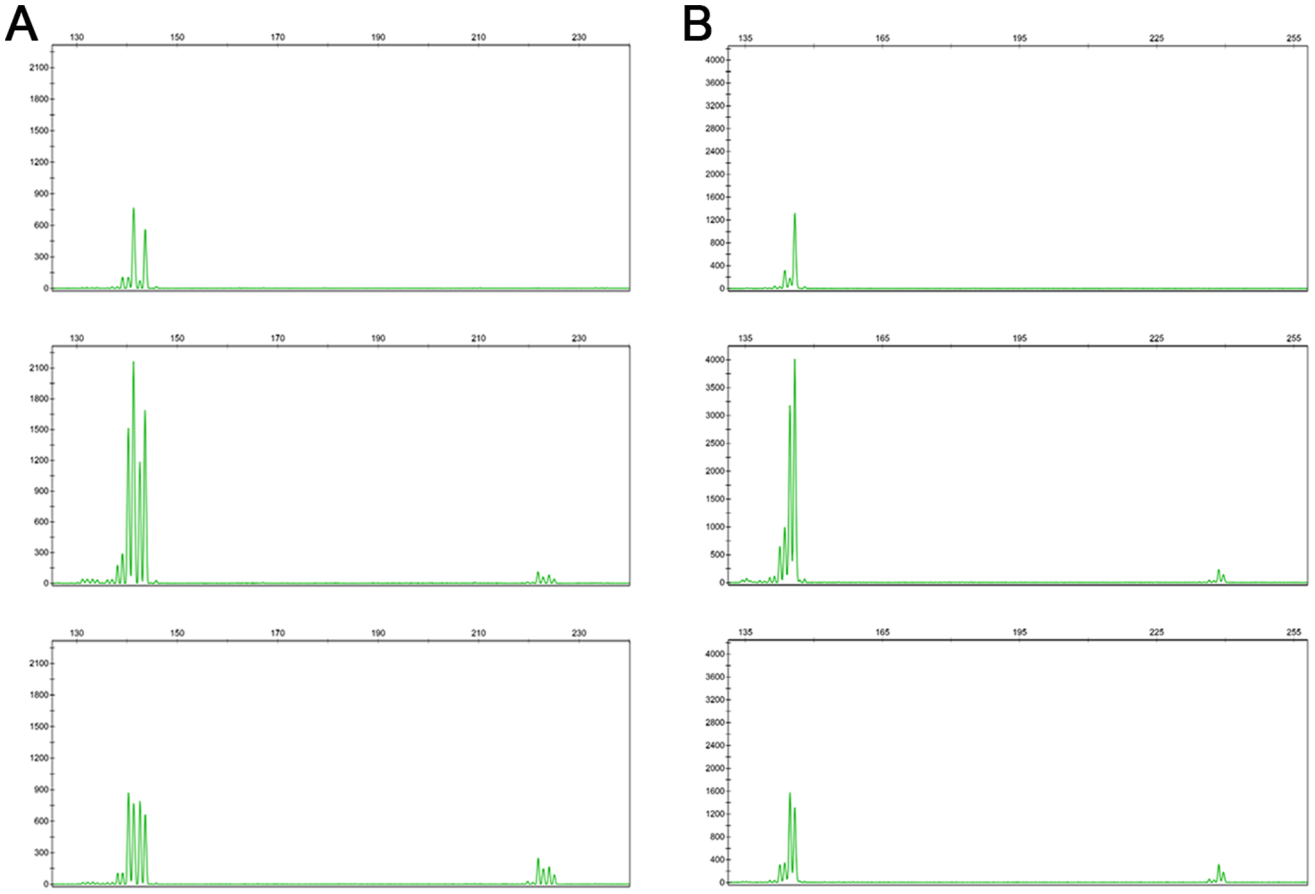
Microsatellite profiles of two DNA samples amplified by loci TkMS010 (left peaks) and TkMS013 (right peaks) through three methods generated on 3130 capillary sequencer. **a**) DNA sample PZ02; **b**) DNA sample PZ04. Each part of the figure contains three screenshots which referred to singleplex, samples amplified by P_E_ and P_F_ then pooled for capillary loading and samples amplified by P_E+F_ using the method described herein.

PCR products of the same DNA samples were grouped in one project to evaluate the quality of allele peaks generated using this method (two multiplex results see Fig 1 and Fig 2, others results of *Tetranychus* species can be found in Figures S1-S38 in Online Resources S1, results of *N. lugens* and *F. occidentalis* can be found in Figures S39–S42 and Figures S43–S46, respectively in Online Resources S2). As expected, the majority of the signals were weaker than the conventional singleplex PCR because all pairs of primers competed for a certain amount of resources, such as templates and enzyme. However, the signals strength was still sufficient to measure the fragment length. The expected microsatellite morphology was observed. No difference in fragment length was observed. For the *T. truncatus*, all the loci were amplified well and showed the same allele size, which was sufficient for studies of population genetics. The DNA sample XZ04 was amplified with loci TUFG55, TUFG57, TUFG87 and TUFG177 using all three methods described above. Results are shown together in Fig. 3 (other results including eight samples of XZ amplified by P_A+B+C_ are available in Figures S25 and S26 in Online Resources S1). For *T. kanzawai*, locus TkMS013 was unable to amplify any DNA samples using any of the normal methods of singleplex PCR. It did work, however, when the multiplex method was used (Fig 4). This result persisted in several trials. This success cannot be attributed to the primers because the primer mixes for multiplex came from primer mix P_S_ (three-primer system for each specific locus) for a single PCR. All the other loci of *T. kanzawai* performed well for all three methods. Another phenomenon was observed at some loci as shown in locus TkMS010 (Fig 4). These loci showed greater signal of stutter peak which adjacent to the true peak. This variation was stable throughout the experiments. Thus, once bins were determined (no matter the stutter peak or the true peak), the alleles calling remained consistent.

Examination of genotyping quality by two populations of *T. truncatus* showed the results to be consistent with those of a previous paper on SSR development [14]. As expected, 2 bp differences in allele size were observed in the loci TUFG130N and TUFG147N as T7 tag was used instead of M13. The quality of the genotype data was confirmed using Micro-Checker 2.2.3. It did not show stuttering or large allelic dropout and the characteristics of loci were consistent with the results of a previous paper and of a structural study that is currently in progress (data not shown). For these eight loci in P_A+B_, the PIC ranged from 0.402 (TUFG146) to 0.790 (TUFG87). The mean number of alleles per locus was 6.5. The observed and expected heterozygosity across the two populations ranged from 0.048 (TUFG146 in XZ) to 0.820 (TUFG57 in SQ) and from 0.091 (TUFG146 in XZ) to 0.752 (TUFG57 in SQ), respectively. Loci TUFG57, TUFG87 and TUFG177 in population XZ deviated from HWE due to heterozygote deficits with null allele frequency >0.120 (0.120–0.230) and inbreeding coefficient >0.206 (0.206–0.364). No departure from HWE was observed at any of locus in the SQ population and there was no indication of any null allele. All inbreeding coefficients were <0.133. All these results demonstrate that all the amplifications took place in a correct and consistent manner when the multiplex method was used.

This multiplex protocol reduces the cost dramatically because it makes full use of the fluorescent dyes. Costs can also be decreased by using Qiagen Multiplex PCR Kits sparingly. Here the PCR final volume was reduced to 5 μL with a buffer concentration of 0.7 X, without compromising performance or specificity [26]. The PCR products were a mixture of several amplifications, which could be diluted and directly mounted on the Sequencer. Here, we presented another suite of universal primers with lower annealing temperature (approximately 52°C) facilitating the modification of existing SSR primers which have annealing temperatures between 52°C and 58°C. By directly comparing the different level of multiplex, we found that most loci in multiplex showed lower signal than in singleplex, which can be reasoned by the competition between loci. However, no significant signal reduction was found between level II and level III. At level II, one locus monopolized one fluorescent dye while at level III one fluorescent dye were shared by at least two loci. Thus multiplex with multiple fluorescent dyes is preferred.

For optimized results the following must be held true: (1) the amount of labeled forward primer: reverse primer: dye-labeled universal primer  = 1∶4∶4. This ratio must hold true for each specific P_S_. Then different P_S_s should be mixed in equimolar amounts before use. When trial results were obtained, the amount of each P_S_ present can be adjusted if required. (2) Loci with different universal tags (different dye colors) can overlap in size. Loci with same dye should be 50 bp apart or even larger (the larger the better). (3) A touchdown PCR protocol can be used if the annealing temperatures of the loci vary only slightly. The last 12 cycles should be run at an annealing temperature of 52°C. (4) The results obtained using this protocol are comparable with each other. When referring to the published allelic length and frequency, the user must subtract from the capillary sequencer 20 bp of the T7 tail sequence (or 18 bp of AP2, M13 and M13R) plus 1 bp of the A overhang appended by HotStarTaq Plus DNA polymerase contained in the Type-it Multiplex PCR Master Mix. (5) If the labeled universal primer amplified non-specific amplifications as a result of homology with the target genome, the user can prepare the primer mix without universal primers. He or she may then pause the latter PCR cycles and add the four universal primers. This option is derived from the method described by de Arruda *et al*. [7]. (6) In principle, this multiplex method with the new set of universal primers can support up to about 20 loci in a single PCR reaction (each dye labeled 5 loci). In practice, 8–12 loci are relatively easy to study if no strong cross-primer or self-complementarity is present. Primer design software such as Multiplex Manager [16] may help.

In conclusion, the new set of universal primers for multiplex genotyping proved to be efficient and reliable in varied species. Usually, using an equal amount of primers (P_S_) in mix can obtain a desirable result with the help of commercial PCR kits. However, primer concentration adjustment is needed especially when loci with large fragment length showed extremely low signal.

## Supporting Information

Online Resources S1 **Original peak pictures of genotyping results of** ***Tetranychus*** **species.**
(RAR)

Online Resources S2 **Original multiplex results of** ***N. lugens*** **and** ***F. occidentalis*** **.**
(RAR)
